# Personalized prostate cancer screening among men with high risk genetic predisposition- study protocol for a prospective cohort study

**DOI:** 10.1186/1471-2407-14-528

**Published:** 2014-07-21

**Authors:** David Margel, Ofer Benjaminov, Rachel Ozalvo, Liat Shavit Grievink, Inbal Kedar, Rinat Yerushalmi, Irit Ben-Aharon, Victoria Neiman, Ofer Yossepowitch, Daniel Kedar, Zohar Levy, Mordechai Shohat, Baruch Brenner, Jack Baniel, Eli Rosenbaum

**Affiliations:** 1 Division of Urology, Rabin Medical Center, Beilinson Campus, Petah-Tikva, Israel; 2 Davidoff Cancer Center, Rabin Medical Center, Beilinson Campus, Petah-Tikva, Israel; 3 Department of Diagnostic Imaging, Rabin Medical Center, Beilinson Campus, Petah-Tikva, Israel; 4 Raphael Recanati Genetic Institute, Rabin Medical Center, Beilinson Campus, Petah-Tikva, Israel

**Keywords:** BRCA, Prostate cancer, Screening, High-risk

## Abstract

**Background:**

Prostate cancer screening among the general population is highly debatable. Nevertheless, screening among high-risk groups is appealing. Prior data suggests that men carrying mutations in the BRCA1& 2 genes may be at increased risk of developing prostate cancer. Additionally, they appear to develop prostate cancer at a younger age and with a more aggressive course. However, prior studies did not systematically perform prostate biopsies and thus cannot determine the true prevalence of prostate cancer in this population.

**Methods:**

This will be a prospective diagnostic trial of screening for prostate cancer among men with genetic predisposition. The target population is males (40–70 year old) carrying a BRCA1 and/or BRCA2 germ line mutation. They will be identified via our Genetic counseling unit. All men after signing an informed consent will undergo the following tests: PSA, free to total PSA, MRI of prostate and prostate biopsy. The primary endpoint will be to estimate the prevalence, stage and grade of prostate cancer in this population. Additionally, the study aims to estimate the impact of these germ line mutations on benign prostatic hyperplasia. Furthermore, this study aims to create a bio-bank of tissue, urine and serum of this unique cohort for future investigations. Finally, this study will identify an inception cohort for future interventional studies of primary and secondary prevention.

**Discussion:**

The proposed research is highly translational and focuses not only on the clinical results, but on the future specimens that will be used to advance our understanding of prostate cancer patho-physiology. Most importantly, these high-risk germ-line mutation carriers are ideal candidates for primary and secondary prevention initiatives.

**Trial registration:**

ClinicalTrials.gov: NCT02053805.

## Background

### The prostate cancer screening debate

Prostate cancer is the most frequently diagnosed cancer and the second leading cause of cancer-related death among men [1]. The most notable feature of prostate cancer diagnosis and staging in the last two decades was the shift from a disease that presented late, either as locally advanced or metastatic tumor, to one that is found in an earlier, often pre-clinical, stage [2]. The exponential increase in the number of cases of early-stage disease has brought with it queries regarding the optimal method of treating these cases. We now question the necessity of diagnosing prostate cancer at an early stage, as earlier detection has not been convincingly associated with improved outcomes and may increase harm [3-5].

This shift may be attributed mainly to the wide-scale use of prostate-specific antigen (PSA) as a screening tool and trans-rectal ultrasound (TRUS)-guided biopsy for diagnosis. At present, most patients with prostate cancer are detected by an elevated serum PSA, leading to trans-rectal biopsy [1,2].

Recently, two large prospective randomized trials designed to test the hypothesis that PSA-based screening would reduce mortality were published. The European Randomized Study of Screening for Prostate Cancer (ERSPC) tested this in Europe [6,7], and the Prostate, Lung Colorectal, and Ovary (PLCO) study [8] tested it in the United States. The European study found that screening was associated with reduced prostate cancer–specific mortality compared with no screening in a subgroup of men aged 55 to 69 years after 9 years (relative risk, 0.80 [95% CI, 0.65 to 0.98]; absolute risk reduction, 0.07 percentage point). The PLCO study found no statistically significant effect after 10 years (relative risk, 1.1 [CI, 0.80 to 1.5]). One of the notable criticisms of the PLCO study is that up to 56% of men in the control arm underwent PSA screening compared with 15% in the European study.

The most common side effects associated with prostatic biopsies are hematospermia, hematuria, fever, hospitalization for prostatitis or urosepsis, and urinary retention [9-11]. Whether the harms of screening are justified by the benefits, in terms of the reported reduction of prostate cancer mortality, is still a very controversial topic. Recently, the U.S. Preventive Service Task Force published a review of the evidence for screening for prostate cancer and made a grade D recommendation against it [3]. On the contrary, the American Society of Clinical Oncology, the American Urological Association and Society of Urological Oncology all state that despite the limitations to the existing data, there is evidence to suggest that men with longer life expectancy may benefit from PSA testing [4,5].

Given the ratio of prostate cancer incidence to prostate-cancer-related mortality, performing routine biopsies for all men would result in many healthy men being labeled as patients unnecessarily. Thus, identifying men at elevated risk of developing potentially life-threatening prostate cancer is a necessity. Men with germ line genetic mutations may represent such a high risk group.

### Genetics and prostate cancer

The pathogenesis of prostate cancer is complex and multifactorial. Consequently, only a limited number of important risk factors for prostate cancer are well accepted. In general, the risk of prostate cancer is increased by African ethnicity, increasing age, and family history. As with other cancers, familial clustering of prostate cancer has been reported. It is now estimated that 5% to 10% of prostate cancer cases are due primarily to high-risk inherited genetic factors or prostate cancer susceptibility genes [12]. Genetic studies suggest seven potential genes are involved in Hereditary Prostate Cancer. Three of these are located on chromosome 1 (HPC1, PCAP and CAPB) and the other four are located on chromosome 17 (HPC2), chromosome 20 (HPC20), chromosome 8 and the X chromosome (HPCX) [13-17]. However, no single susceptible gene is, by itself, responsible for a large portion of familial prostate cancers.

There are also several genetic conditions associated with an increased risk of prostate cancer. Hereditary breast and ovarian cancer (HBOC) syndrome and Lynch syndrome are the most common ones. HBOC is associated with mutations in the BRCA1 and/or BRCA2 (BRCA stands for BReast CAncer). HBOC is most commonly associated with an increased risk of breast and ovarian cancer in women. However, men with HBOC also have an increased risk of breast cancer and prostate cancer [12].

### BRCA and prostate cancer

BRCA1 and BRCA2 are tumor suppressor genes. In normal cells, BRCA1 and BRCA2 help ensure DNA stability and help prevent uncontrolled cell growth. Genetic instability is a characteristic of BRCA1/2 deficient cells that leads to an accumulation of genomic and post genomic abnormalities. Mutations of these genes are linked to the development of HBOC. However, these genetic mutations do not affect only woman. Several studies have reported that the risk of prostate cancer is higher among men carriers of both BRCA 1 and 2 [15-20]. The results from the Breast Cancer Linkage Consortium (BCLC) showed a RR of 4.65 of prostate cancer in male BRCA2 mutation carriers (RR 7.33 below the age of 65 years) and 1.07 in BRCA1 carriers (with a RR of 1.82 for men under 65 years old) [21,22]. Recent studies have suggested that the risk for male BRCA1 mutation carriers may be lower than previous estimates and that BRCA2 mutation carriers may have a significantly higher RR of 23-fold at age 60 [23,24]. Furthermore, BRCA mutations may be linked not only to susceptibility to prostate cancer, but also to the aggressiveness of the disease [17-20].

The largest study to date was recently published at JCO [20]. This study analyzed the tumor features and outcomes of 2,019 patients with prostate cancer (18 BRCA1 carriers, 61 BRCA2 carriers, and 1,940 non-carriers). Germline BRCA1/2 mutations were associated with higher grade, stage, nodal involvement and metastasis at diagnosis. Prostate specific survival was significantly longer among non-carriers compared to BRCA carriers (15.7 v 8.6 years). Subgroup analyses confirmed the poor outcomes in BRCA2 patients, whereas the role of BRCA1 was not well defined due to the limited size and follow-up in this subgroup.

To summarize, prostate cancer tends to be an indolent cancer mainly affecting older men. However, individuals carrying germline mutations such as BRCA 1/2 are not only at increased risk of developing prostate cancer, but there is evidence suggesting that this high-risk group may develop prostate cancer at a younger age with a more aggressive phenotype. Clearly, in these subjects there is a role for a personalized screening approach.

### Objectives

The purpose of this study is to:

1) Determine the prevalence and severity of prostate cancer among BRCA carriers.

2) Determine the accuracy and cost-effectiveness of different screening options for prostate cancer in this group (PSA, free to total PSA, prostate MRI).

3) Determine the prevalence and severity of benign prostatic hypertrophy and lower urinary tract symptoms among this group.

4) Establish a biobank of this unique population.

## Methods/design

### Study design

This is a prospective diagnostic trial of screening for prostate cancer among men with high genetic predisposition (BRCA1\2) to estimate the incidence of prostate cancer, and the accuracy and cost effectiveness of different screening modalities in this population. Additionally, this study aims to estimate the impact of these germline mutations on BPH and LUTS. Furthermore, this study aims to create a bio-bank of tissue, urine, and serum of this unique cohort for future investigations. Finally, this study will identify an inception cohort for future interventional studies of primary and secondary prevention.

### Study population

The target population is males carrying germline mutations in BRCA1 or BRCA2. Eligible men, carriers of one or more of the above mentioned mutations, will be identified through collaboration and under supervision of the genetic clinic in Beilinson hospital.

Individuals expressing interest in taking part in the study will be contacted by the research team and an initial appointment will be scheduled. The individual will be given the option to participate in the MRI and the prostatic biopsy. Fully informed written consent will be sought before collecting any research samples.

Inclusion criteria

• Male carrier of mutation in BRCA 1\2.

• Age 40–70 years.

• WHO performance status 0–2 (Additional file 1).

• No previous history of prostate cancer.

• No previous prostate biopsy.

• Absence of any psychological, familial, sociological or geographical situation potentially hampering compliance with the study protocol and follow-up schedule.

• Individuals that cannot undergo the MRI exam due to high creatinine level or claustrophobia will be excluded from the MRI part.

• Informed written consent must be sought according to ICH/EU GCP, before subject I inclusion.

Exclusion criteria

• Previous cancer with a terminal prognosis of less than five years.

• Previous prostate cancer.

### Primary endpoint

To determine the prevalence, stage, and pathology of screen-detected prostate cancer in BRCA1/BRCA2 founder mutation carriers.

### Secondary endpoints

1. To test the accuracy (sensitivity, specificity, positive and negative predictive value) of different screening tests (PSA, free to total PSA, prostate MRI) in detecting prostate cancer among men with genetic predispositions.

2. To test the accuracy (sensitivity, specificity, positive and negative predictive value) of different screening tests (PSA, free to total PSA, prostate MRI) in detecting clinically significant prostate cancer among men with genetic predispositions.

3. To establish the cost effectiveness of different screening tests (PSA, free to total PSA, prostate MRI) in detecting prostate cancer and clinically significant prostate cancer among men with genetic predispositions.

4. To determine the impact of these genetic mutations on lower urinary tract symptoms (International Prostate Symptoms Score - IPSS, flow and post void urine residual) and BPH (trans-rectal US prostate size).

5. To characterize the genomic and biological profiles in samples from these mutation carriers and characterize changes related to prostate cancer.

### Study procedures

**Figure 1:**
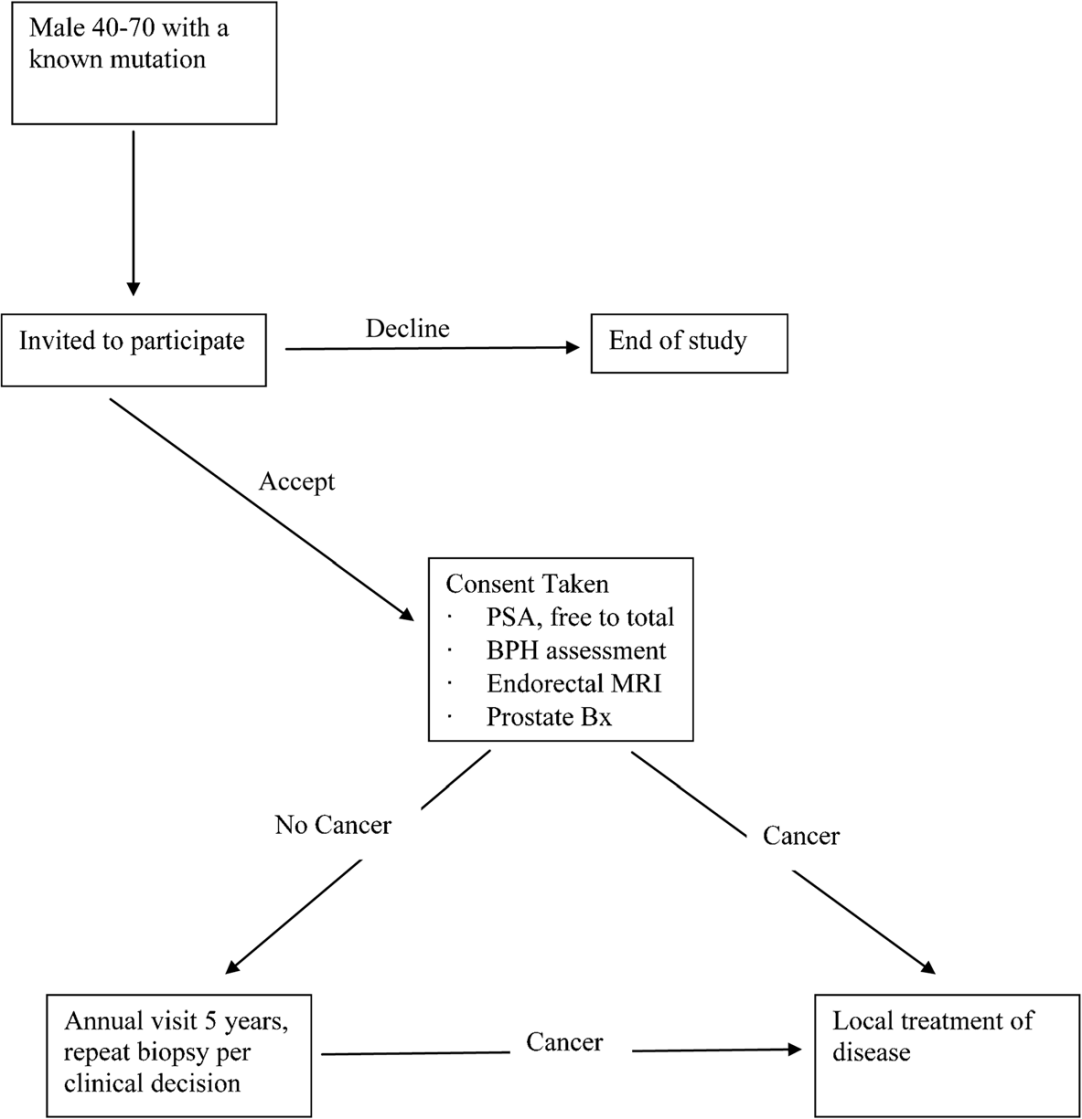
CONSORT diagram.

The study flowchart is presented in Figure 1; study timeline is presented in Additional file 2.

#### First round of screening

The following investigations will be performed in the first visit after informed consent was obtained.

1. Each patient will provide 50 ml blood sample for PSA and free to total PSA tests. The serum will be stored for future investigations.

2. Each patient will be asked to complete the IPSS questionnaire - a validated lower urinary tract symptom questionnaire (Additional file 3).

3. DRE followed by a urine sample will be provided for storage (Additional file 4). Creatinine level will be checked.

4. Each patient’s urinary flow and post void residual will be measured. The post void residual will be recorded by using ultrasound.

5. Subjects will undergo a multi-parametric prostate MRI using the Ingenia 3.0Tesla with the following protocol:

• Axial, T2-weighted and T1-weighted turbo spin echo imaging will be performed over the pelvic region. 40 slices acquired with a thickness/gap of 5.0/1.0 mm, FOV AP 300 mm × RL 300 mm × FH 239 mm. For the T2-weighted scan, a SPAIR adiabatic fat suppression will be applied. The TR/TE 3800/80ms, the acquisition matrix 272 × 209, turbo factor 19, NEX = 1 and the SENSE parallel imaging factor 2.0 for an imaging time of 2:17 min. For the T1-weighted scan, the TR/TE s 567/8.0 ms, the acquisition matrix 332 × 288, turbo factor 8, NEX = 1 and the SENSE parallel imaging factor 2.0 for an imaging time of 2:57 min.

• For prostate imaging, T2-weighted turbo spin-echo images obtained in three orthogonal planes (axial, sagittal and coronal). The TE 120 ms, and the TR set the shortest possible (4000 – 6000 ms). The remaining scan parameters of T2-weighted images are presented in Table 1.

**Table 1:** Additional scan parameters of T2-weighted images

**Slice Direction**	**No. of slices**	**Slice width/gap (mm)**	**Turbo factor**	**FOV mm**		**Nex**	**Acq matrix**	**WFS (pixels)**	**Scan time (min)**
				**AP**	**RL**				
Axial	30	2.5/0.25	24	160	160	1	268 × 219	1.5	3:20
Coronal	20	3.0/0.3	26	180	180	2	300 × 248	1.0	4:40
Sagittal	20	3.0/1.0	26	180	180	2	300 × 252	1.0	4:17

• Axial DWI obtained using a modified Stejskal-Tanner spin-echo echo-planar imaging (EPI) sequence with the following parameters: TR/TE 4000/88 ms; flip angle 90; NEX = 6; *b*-values 0, 100, 1000 and 1500 s/mm^2^; matrix (M × P) 116 × 101; FOV AP 300 mm × RL 350 mm × FH 56 mm. 17 slices acquired with a thickness/gap of 3.0/0.3 mm covering the entire prostate and seminal vesicles. A SPAIR adiabatic fat suppression applied and a SENSE parallel imaging factor of 2.4 used for a scan time of 4:04 min.

• Contrast uptake by the prostate is monitored using a 3D dynamic T1-weighted, single-shot, turbo field echo sequence. The TR/TE 3.1/1.45 ms, flip angle 10°; NEX = 2; matrix 124 × 124; FOV AP 200 mm × RL 200 mm × FH 72 mm; 41 slices acquired with a thickness of 3.5 mm sampled at 1.75 mm. A SPAIR adiabatic fat suppression pulse applied. The dynamic scan time is 23.1 sec, and a total of 10 dynamics are acquired for a total scan time of 3:54 min.

• All MRIs will be done prior to prostate biopsy to minimize artifacts, and enable US-MRI fusion biopsies [25,26] if a lesion is detected. Prior to the examination, glucagon will be administered intramuscularly to diminish artifacts from bowel peristalsis. Diagnostic features for malignancy will be a low T2 signal in the peripheral zone, a relatively low ADC calculated from DWI, early enhancement and washout on DCE MRI. For the transitional zone a poorly defined nodule that distorts the normal architecture and concordant abnormalities on DWI and DCE MRI will be considered suspicious for malignancy. The MRI will be reported on a 5 point Likert Scale (Additional file 5). If creatinine level is above 1.8 mg/dl, the patient will be excluded from the MRI part of the study.

6. Subjects, who agree to prostate biopsies, will undergo a 12 core Trans-rectal prostatic biopsy for diagnostic purposes. Prostate biopsies will be performed by a single expert Uro-Oncologist (DM) in accordance with the specifications and procedures detailed in Additional file 6. An attempt will be made to collect at least a minimum core length of 1 centimeter on each biopsy core, when possible, in order to allow for a complete analysis. It is recognized that in some areas of the prostate this may be difficult but it is encouraged to obtain as long a core as possible. Cores with any amount of prostate tissue are counted as part of the 12-core schema. If no tissue is obtained in a biopsy core, the core may be repeated. In the event of a focal lesion on MRI, we will continue to follow the biopsy diagram for core sample location and if the focal lesion is not part of the standard 12-cores, we will obtain an additional sample of the focal lesion using MRI-US fusion system [25,26]. The location of the biopsies will be labeled and sent to pathology. A single dedicated Uro-pathologist will review all pathological specimens.

The reason to offer all subjects a prostate biopsy irrespective of their PSA is that there is very limited evidence that PSA screening is useful among patients with BRCA mutations. Furthermore, results from the PCPT trial, which offered prostate biopsy to all subjects (independent of their PSA level), demonstrated that as many as 15% of men with a PSA value less than 3.0 ng/mL had prostate cancer and that 15% of these cancers were high grade. These results may be even more pronounced in a population of high- risk men with germline mutations. Moreover, data is accumulating that prostate cancer may be more aggressive among this subset of patients. Finally, the risk of serious side effects from prostate biopsies is minimal among a young and healthy population. We therefore believe that prostate biopsies are essential.

7. After results of the PSA, MRI, and pathology are available a visit will be scheduled with an urologist to determine further follow-up or treatment. If the biopsy is normal subjects will enter an annual PSA-based screening (for the first 5 years). If High Grade PIN is identified at biopsy, it is recommended that the biopsy is repeated after 6 months. If Atypical Small Acinar Proliferation (ASAP) is identified at biopsy, it is recommended that the biopsy is repeated after 3 months. The urologist will also review the results of the LUTS assessment and will offer an individualized treatment or follow up.

#### On annual review

Medical and family history will be updated, and then each subject will undergo PSA testing, and plasma and urine storage (Additional file 4). A decision to repeat prostate biopsy will be made based on clinical grounds (i.e. an abnormal PSA measurement)

#### If prostate cancer is diagnosed

The staging and further investigation of the disease is directed by the uro-oncology unit. Management is based on the pathology information and discussion between the Uro-oncologist and patient.

The following information will be recorded:

• Clinical T stage.

• Gleason grade of biopsy (primary and secondary).

• Volume of cancer on biopsy (i.e. the number of cores involved and percent of each involved core).

• Treatment and management plan (i.e. active surveillance, surgery, or radiation).

• Radiological TNM stage- if clinically appropriate results of CT scan + bone scan.

• Histopathology report.

• Annual follow-up will be offered to patients after prostate cancer diagnosis regardless of treatment center (we realize that not all patients will receive treatment at Rabin Medical Center).

• Progression, for those choosing active surveillance, and Biochemical recurrence rate, for those choosing surgery or radiation, will be monitored.

• Survival will also be monitored, but the number of prostate cancer deaths is unlikely to be sufficient for statistical analysis.

### Potential adverse events

As detailed earlier, we believe that a transrectal ultrasound and biopsy should be carried out on all subjects. This procedure will be done under local anesthesia; however, it is uncomfortable and associated with the following risks:

• Painful or difficult voiding- this may appear in 13% of patients, and usually lasts several hours.

• Hematuria- this is usually minimal, and may appear in 11% of patients and last for several hours.

• Septicemia- this is the most serious potential adverse effect. It may appear between 0.5-3% of patients. It usually occurs following repeated biopsies and among immune suppressed or older subjects. The target population in our study is young and non-had prior prostate biopsy (see inclusion/exclusion criteria). We therefore expect a low rate of septicemia. However, we will administer prophylactic Abx, as per protocol, and will ask any subject with fever or chills after the procedure to seek medical attention immediately.

• Acute urinary retention – this is very rare (0.1-1% of patients), and usually occurs among elderly subjects [9-11].

For this reason, subjects will be followed carefully and be able to contact the urology department in case of problems.

### Removal from the study

Subjects may withdraw from the study at any time, if they so wish, without giving a reason. Data will be censored for participants who develop prostate cancer or are too unwell to attend screening.

### Statistics

#### Primary analysis

The primary aim of this study is to estimate the true prevalence of prostate cancer among men with genetic predisposition. Since our study will offer a prostate biopsy to all subjects upon entry we will avoid “verification bias” associated with PSA based screening. However, this fact prevents us from including a control group, as we feel it may be unethical to offer non-carrier such a work-up. Therefore, we will use descriptive statistics only to report the primary outcome stratified by mutation. The fact that we are not including a control group also prevents us from providing a sample size calculation. We will perform a preliminary analysis of our data after 220 patients.

### Secondary analysis

1. Descriptive statistics stratified by mutation will be used to report stage and grade of prostate cancer.

2. Sensitivity, specificity, positive and negative predictive value of: PSA, free to total PSA, prostate MRI in detecting prostate cancer among men with genetic predispositions will be calculated.

3. A Receiver Operating Curve (ROC), and calibration and decision curve analyses will be performed for each of the aforementioned screening tests. The threshold for biopsy for each test will be calculated based on any prostate cancer detected as well as clinically significant cancer detected (for the purpose of this study any cancer with a Gleason sum equal or higher than 7 will be considered clinically significant).

4. We will use a Markov model to establish the cost effectiveness of different screening tests (PSA, free to total PSA, prostate MRI) in detecting prostate cancer and clinically significant prostate cancer among men with genetic predispositions.

5. Descriptive statistics stratified by mutation will be used to report the impact of these genetic mutations on lower urinary tract symptoms (IPSS, flow and post void urine residual) and BPH (trans-rectal US prostate size).

#### Harms

The conduct of the study will comply with all Israeli Health Ministry safety reporting requirements. All adverse experience reports must include the patient number, severity of reaction (mild, moderate, severe), relationship to study drug (probably related, unknown relationship, definitely not related), date and time of administration of test medications and all concomitant medications, and medical treatment provided. The principal investigator is responsible for evaluating all adverse events to determine whether criteria for “serious adverse events”, as defined above, are present. Investigators must notify the Rabin Medical Center Institutional Review Board (IRB) of all SAE and file the report in the regulatory study binder. Documentation from the IRB of receipt of these reportable events must be kept on file. A clear description of the suspected reaction should be provided along with an assessment as to whether the event is related to the study.

#### Auditing

The Data Safety and Monitoring Board (DSMB) will perform a planned audit 1.5 years from initiating the study. Regulatory agencies may also conduct a regulatory inspection of this study. Such audits/inspections can occur at any time during or after completion of the study. If an audit or inspection occurs, the investigator and institution agree to allow the auditor/inspector direct access to all relevant documents and to allocate his/her time and the time of his/her staff to the auditor/inspector to discuss findings and any relevant issues.

### Ethical matters

The study is conducted according to the principles of the declaration of Helsinki (2008) and the Medical Research involving Human Subjects Act (WMO), and has been approved by the Rabin Medical Center IRB. Written informed consent will be obtained from all patients before enrolment.

## Discussion

The only ongoing study specifically designed to test PSA screening among BRCA Carriers is the IMPACT study [20,27]. The IMPACT study, led by Professor Rosalind Eeles, is a multicenter observational study of screening for prostate cancer. In this study, male BRCA carrier’s, 40 to 69 years old, are offered annual PSA testing and the threshold for prostatic biopsy is any PSA higher than 3 ng/ml. Recently, the first round of screening was published [25]. In this study, a total of 199 men (8%) presented with PSA >3.0 ng/ml, 162 biopsies were performed, and 59 PCas were diagnosed (18 BRCA1 carriers, 10 BRCA1 controls; 24 BRCA2 carriers, 7 BRCA2 controls); 66% of the tumors were classified as intermediate- or high-risk disease. A final report is expected in 2018.

The proposed study, although targeting a similar population, is different in several important aspects. First, in the IMPACT study only patients with an elevated PSA level are offered a prostate biopsy. In a preliminary report on the first round of screening, only 8% had a biopsy [25]. The fact that only such a small percentage of patients were offered a biopsy, and that it was offered based on PSA levels may cause a “verification-bias” [27,28].

Verification bias is a type of measurement bias. This bias occurs when results of a screening test (i.e. PSA) affect whether a diagnostic test (i.e. prostate biopsy) is used. Results from the PCPT trial [29], which offered prostate biopsy to all subjects (independent of their PSA level), demonstrated that as many as 15% of men with a PSA value less than 3.0 ng/mL had prostate cancer and that 15% of these cancers were high grade. These results may be even more pronounced in a population of high-risk men with germline mutations. To eliminate this bias, we will offer a prostate biopsy to all men included in the study regardless of the PSA level.

The second notable difference between our study and the IMPACT study is the target population. Our institution is located in Israel where the predominant BRCA mutations are the three founder mutations. There is evidence to suggest a genotype-phenotype difference among BRCA founder mutation carriers compared to other germ-line mutations [30,31]. In addition, since 2% of Ashkenazi Jews carry one of the founder mutations we will have enough power to detect a difference in prostate cancer parameters between the three mutations [31].

We also propose a more comprehensive preliminary screening compared to the IMPACT study. In our study the initial screening will include: DRE, PSA free to total PSA, a multi-parametric prostate MRI and a trans-rectal ultra-sound guided prostate biopsy. Thus, we will be able to determine the value of MRI screening, and determine the best screening modality. In breast cancer for instance, female BRCA carriers are advised to complete both mammography and MRI [32]. Each of these screening tests provides valuable information and both are needed. The same may be true in male carriers.

We will also include a Benign Prostatic Hyperplasia (BPH) assessment: the validated International Prostate Symptom Score (IPSS), trans-rectal US assessment of prostate size, urine flow and residual. To the best of our knowledge, there is no data regarding the impact of these mutations on BPH. Since there is a prospect of identifying new biomarkers in this population we will also store whole blood, lymphocytes, serum, plasma, urine, and prostate tissue for future studies on all subjects.

In summary, this study protocol offers male BRCA carriers a complete prostate assessment including both cancer and BPH measurements. This is a very unique population and unlike most places in the world, Israel has a large proportion of BRCA carriers, as 2% of the Ashkenazi Jews are BRCA carriers. So far, male BRCA carriers are largely ignored. In biological research, the study of an unusual or rare event sometimes allows the identification of key features of more common forms. A good example of this has been the cloning and characterization of tumor suppressor genes in rare familial renal cell cancer. Data from this study will be used not only to understand the link between BRCA and prostate cancer risk. We will also store tissue, whole blood, serum, and urine for future studies that may aid our understanding of prostate cancer patho-physiology.

## Competing interests

We have no financial conflict of interest to declare.

## Authors’ contributions

DM conceived the study and participated in its design, and was involved in drafting the protocol. LSG and RO participated in the design of the study and were involved in drafting the protocol. OB, IK, RY, IBA, VN, OY, DK, ZL, MS, BB, JB, and ER participated in the design of the study and provided critical revisions to the protocol. All authors read and approved the final manuscript.

## Pre-publication history

The pre-publication history for this paper can be accessed here:

http://www.biomedcentral.com/1471-2407/14/528/prepub

## Supplementary Material

Additional file 1 WHO performance status. Click here for file

Additional file 2 Study time line. Click here for file

Additional file 3 IPSS questionnaire. Click here for file

Additional file 4 Guidelines for sample collection and storage. Click here for file

Additional file 5 Likert scale for prostate MRI. Click here for file

Additional file 6 Scheme for prostate biopsy. Click here for file
