# Supplementary material for: Personalized prostate cancer screening among men with high risk genetic predisposition- study protocol for a prospective cohort study
Source: BMC Cancer. 2014 Jul 21;14:528. doi: 10.1186/1471-2407-14-528 (PMC4223504; doi:10.1186/1471-2407-14-528)
Supplement: Additional file 1 — WHO performance status. [file 1471-2407-14-528-S1.doc]

**Additional file 1: WHO Performance status**

| Grade | Explanation of activity |
| --- | --- |
| 0 | Fully active, able to carry on all pre-disease performance without restriction |
| 1 | Restricted in physically strenuous activity but ambulatory and able to carry out work of a light or sedentary nature, e.g., light house work, office work |
| 2 | Ambulatory and capable of all self-care but unable to carry out any work activities. Up and about more than 50% of waking hours |
| 3 | Capable of only limited self-care, confined to bed or chair more than 50% of waking hours |
| 4 | Completely disabled. Cannot carry on any self-care. Totally confined to bed or chair |
| 5 | Dead |
