# Supplementary material for: Personalized prostate cancer screening among men with high risk genetic predisposition- study protocol for a prospective cohort study
Source: BMC Cancer. 2014 Jul 21;14:528. doi: 10.1186/1471-2407-14-528 (PMC4223504; doi:10.1186/1471-2407-14-528)
Supplement: Additional file 2 — Study time line. [file 1471-2407-14-528-S2.doc]

**Additional file 2: Study time line**

|  | year 1 | | | | | | year 2 | year 3 | year 4 | year 5 | year 6 |
| --- | --- | --- | --- | --- | --- | --- | --- | --- | --- | --- | --- |
| TimePoint | t-0 | t-1 | t-2 | t-3 | t-4 | t-5 | t-6 | t-7 | t-8 | t-9 | t-10 |
|  |  |  |  |  |  |  |  |  |  |  |  |
| **Patient Enrollment:** |  |  |  |  |  |  |  |  |  |  |  |
| eligibility screen | X |  |  |  |  |  |  |  |  |  |  |
| WHO performance test | X |  |  |  |  |  |  |  |  |  |  |
| Informed consent | X |  |  |  |  |  |  |  |  |  |  |
| Mutation carries test | X |  |  |  |  |  |  |  |  |  |  |
| consult meeting |  | X |  |  |  |  |  |  |  |  |  |
| **INTERVENTIONS:** |  |  |  |  |  |  |  |  |  |  |  |
| family tree |  |  | X |  |  |  |  |  |  |  |  |
| PSA test |  |  | X |  |  |  | X | X | X | X | X |
| free PSA test |  |  | X |  |  |  | X | X | X | X | X |
| plasma and serum sample storage |  |  | X |  |  |  | X | X | X | X | X |
| IPSS Questionnaire |  |  | X |  |  |  |  |  |  |  |  |
| DRE test |  |  | X |  |  |  |  |  |  |  |  |
| urine sample storage |  |  | X |  |  |  | X | X | X | X | X |
| urinary flow measured |  |  | X |  |  |  |  |  |  |  |  |
| post void residual measured |  |  | X |  |  |  |  |  |  |  |  |
| Creatinine test |  |  | X |  |  |  |  |  |  |  |  |
| endorectal coil prostate MRI |  |  |  | X |  |  |  |  |  |  |  |
| trans-rectal Biopsy |  |  |  |  | X |  |  |  |  |  |  |
| **ASSESSMENTS:** |  |  |  |  |  |  |  |  |  |  |  |
| consultation meeting |  |  |  |  |  | X |  |  |  |  |  |
