# Supplementary material for: Personalized prostate cancer screening among men with high risk genetic predisposition- study protocol for a prospective cohort study
Source: BMC Cancer. 2014 Jul 21;14:528. doi: 10.1186/1471-2407-14-528 (PMC4223504; doi:10.1186/1471-2407-14-528)
Supplement: Additional file 3 — IPSS questionnaire. [file 1471-2407-14-528-S3.doc]

**Additional file 3: IPSS Questionnaire**

|  | Not at all | Less than 1 time in 5 | Less than half the time | About half the time | More than half the time | Almost always | **Your score** |
| --- | --- | --- | --- | --- | --- | --- | --- |
| Incomplete emptying Over the past month, how often have you had a sensation of not emptying your bladder completely after you finish urinating? | 0 | 1 | 2 | 3 | 4 | 5 |  |
| Frequency Over the past month, how often have you had to urinate again less than two hours after you finished urinating? | 0 | 1 | 2 | 3 | 4 | 5 |  |
| Intermittency Over the past month, how often have you found you stopped and started again several times when you urinated? | 0 | 1 | 2 | 3 | 4 | 5 |  |
| Urgency Over the last month, how difficult have you found it to postpone urination? | 0 | 1 | 2 | 3 | 4 | 5 |  |
| Weak stream Over the past month, how often have you had a weak urinary stream? | 0 | 1 | 2 | 3 | 4 | 5 |  |
| Straining Over the past month, how often have you had to push or strain to begin urination? | 0 | 1 | 2 | 3 | 4 | 5 |  |

|  | None | 1 time | 2 times | 3 times | 4 times | 5 times or more | **Your score** |
| --- | --- | --- | --- | --- | --- | --- | --- |
| Nocturia Over the past month, many times did you most typically get up to urinate from the time you went to bed until the time you got up in the morning? | 0 | 1 | 2 | 3 | 4 | 5 |  |
| Total IPSS score | | | | | | |  |
