# Supplementary material for: Personalized prostate cancer screening among men with high risk genetic predisposition- study protocol for a prospective cohort study
Source: BMC Cancer. 2014 Jul 21;14:528. doi: 10.1186/1471-2407-14-528 (PMC4223504; doi:10.1186/1471-2407-14-528)
Supplement: Additional file 4 — Guidelines for sample collection and storage. [file 1471-2407-14-528-S4.doc]

**Additional file 4: Guidelines for sample collection and storage**

For all samples blood should be drawn:

1. Prior to any manipulation of the prostate
2. At least 24h following ejaculation (if within 24h the time should be noted)
3. 6 weeks after resolution of prostatitis

Details that must be record for each sample:

1. The tube used to collect the sample (should include full details of tube type and Manufacturer)
2. All sample manipulations e.g.

- Time of blood draw
- Time and temperature of centrifugation (where appropriate)
- Time and temperature of storage

**Samples to be collected:**

- Please note that ideally all samples should be processed and frozen as soon as possible on the day that they were taken.
- If samples cannot be processed on the day then samples should be processed in the lab chronologically.
- All blood tubes should be gently inverted (10-15 times) before being placed in the centrifuge.
- The samples will be stored in a -80 freezer at the surgical room, 3'Th floor, Belinson hospital.

1. **Serum collection**

10ml serum will be collected in a universal plastic container. The samples should be transferred to a –80°C freezer as soon as possible.

**2. Urine collection**

30ml urine will be collected in a universal plastic container. The samples should be transferred to a –80°C freezer as soon as possible.
