# Supplementary material for: Personalized prostate cancer screening among men with high risk genetic predisposition- study protocol for a prospective cohort study
Source: BMC Cancer. 2014 Jul 21;14:528. doi: 10.1186/1471-2407-14-528 (PMC4223504; doi:10.1186/1471-2407-14-528)
Supplement: Additional file 5 — Likert scale for prostate MRI. [file 1471-2407-14-528-S5.doc]

**Additional file 5: Likert scale for prostate MRI**

- Score 1 = Clinically significant disease is highly unlikely to be present
- Score 2 = Clinically significant cancer is unlikely to be present
- Score 3 = Clinically significant cancer is equivocal
- Score 4 = Clinically significant cancer is likely to be present
- Score 5 = Clinically significant cancer is highly likely to be present
