# Supplementary material for: Personalized prostate cancer screening among men with high risk genetic predisposition- study protocol for a prospective cohort study
Source: BMC Cancer. 2014 Jul 21;14:528. doi: 10.1186/1471-2407-14-528 (PMC4223504; doi:10.1186/1471-2407-14-528)
Supplement: Additional file 6 — Scheme for prostate biopsy. [file 1471-2407-14-528-S6.doc]

**Additional file 6: Scheme for prostate biopsy**

All biopsies will be performed by a single expert Uro-Oncologist according to the following scheme.


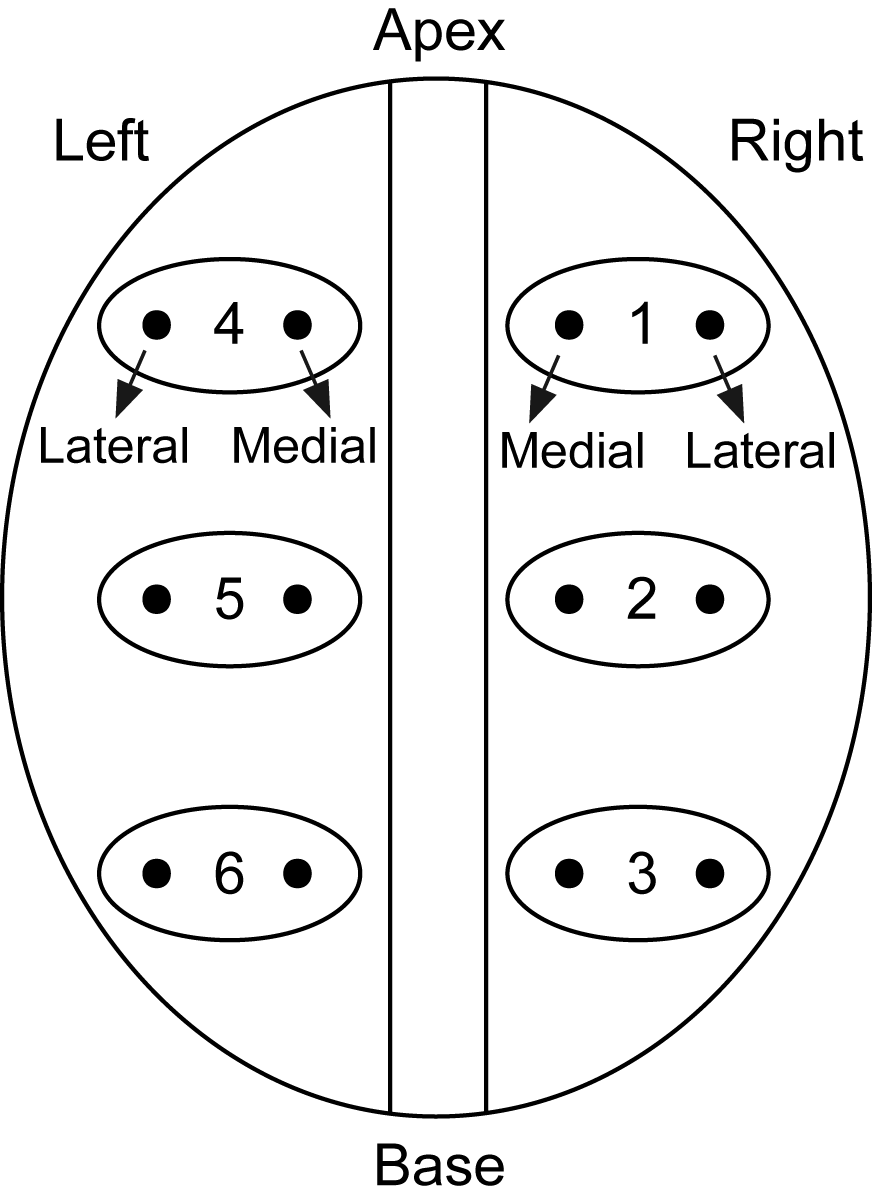


1. Right apex
2. Right median
3. Right base
4. Left apex
5. Left median
6. Left base

If a nodule is identified outside the scheme an additional biopsy will be taken. All cores will be sent and labeled separately to the pathologist.
